# Variability of NOR patterns in European water frogs of different genome composition and ploidy level

**DOI:** 10.3897/CompCytogen.v11i2.10804

**Published:** 2017-04-18

**Authors:** Anna Zaleśna, Maria Florek, Mariusz Rybacki, Maria Ogielska

**Affiliations:** 1 Department of Evolutionary Biology and Conservation of Vertebrates, Institute of Environmental Biology, University of Wrocław, Poland; 2 Department of Zoology, Kazimierz Wielki University, Al. Ossolińskich 12, 85–067, Poland

**Keywords:** *Pelophylax esculentus* complex, hybridogenesis, triploidy, NOR inheritance

## Abstract

We studied water frogs from a complex composed of two species: *Pelophylax lessonae* (Camerano, 1882) (genome LL, 2n = 26) and *P. ridibundus* (Pallas, 1771) (RR, 2 = 26), and their natural hybrid *P. esculentus* (Fitzinger, 1843) of various ploidy and genome composition (RL, 2n = 26, and RRL or RLL, 3n = 39). Tetraploids RRLL were found (4n = 52) in juveniles. We applied cytogenetic techniques: AgNO_3_, chromomycin A_3,_ PI and fluorescent *in situ* hybridization with a 28S rDNA probe. Results obtained by silver staining corresponded well with those stained with CMA_3_, PI and FISH. As a rule, NORs are situated on chromosomes 10. The number of Ag-NORs visible on metaphase plates was the same as the number of Ag-nucleoli present in interphase nuclei of the same individual. In all analyzed metaphases, NORs exhibited variations in size after AgNO_3_ and CMA_3_ stainings. Sixty-six individuals (out of 407 analyzed) were polymorphic for the localization and number of NORs. Fifty-one diploids had NORs only on one chromosome of pair 10. Three triploids (LLR and RRL) displayed two NORs, and two other triploid RRL individuals displayed one, instead of expected three NORs. In ten individuals extra NORs were detected on chromosomes other than 10 (chromosomes 2 and 9).

## Introduction

Nucleolus organizer regions (NORs) are sites of nucleoli formation owing to the presence of genes (rDNA) coding for 18S rRNA, 5.8S rRNA and 28S rRNA. They are the only genes that can be recognized in genomes on the basis of chromosome structure and thereby are useful as cytogenetic markers. These chromosomes differ from others by the presence of secondary constrictions where NORs are situated. The number and position of NORs are species specific, although inter-individual variability of these regions has also been observed within species. In anuran amphibians, NORs detected by silver staining (Ag-NOR) revealed that most species, both from primitive and derived families, have only one pair of NOR-bearing chromosomes in their diploid karyotypes (Schmid 1982, King et al. 1990, Birstein 1984, Vitelli et al. 1982, Bruschi et al. 2012, Carvalho et al. 2014). The localization of NORs is conservative, *i.e.* they are almost always located intercalary or proximally to the centromeres or close to telomeres or related to other regions rich in heterochromatin (Iizuka et al. 2013). Exceptions to this rule may suggest chromosomal rearrangements that have occurred in NOR-carrying chromosome segments during evolution (Schmid 1982). Variations of Ag-NORs may be connected with different expression of rRNA genes during the preceding interphase (Reeder 1990). Using silver staining, only active NORs are identified and thereby the actual sites of rDNA transcription should be verified by more specific methods, such as fluorescence *in situ* hybridization (FISH) with use of 18S or 28S rDNA probes that unequivocally indicate the rDNA loci (Iizuka et al. 2013). The variability of NORs can also reflect geographical karyotypic variations. In the endemic frog *Proceratophrys boiei*, NORs were located on different chromosome pairs, depending on geographical region of its distribution (Amaro et al. 2012). Similar results were described in *Physalaemu olfersii* (Silva et al. 2000), *Hypsiboas* (Carvalho et al. 2014), *Physalaemus cuvieri* (Quinderé et al. 2009), and in the Jefferson salamanders *Ambystoma jeffersonianum* (Bi et al. 2009).

In water frogs, NORs are located in secondary constrictions on long arms of chromosome pair 10, as observed after AgNO_3_ staining (Schmid 1982, Koref-Santibanez and Günther 1980, Heppich et al. 1982, Vitelli et al. 1982), chromomycin A_3,_ and FISH with 18S+28S probes (Spasič-Bošković et al. 1999, Martirosyan and Stepanyan 2009).

Central European water frogs form a complex composed of two species: *Pelophylax lessonae* (Camerano, 1882) (genome LL, 2n = 26) and *P. ridibundus* (Pallas, 1771) (RR, 2 =26), and their natural hybrid *P. esculentus* (Fitzinger, 1843) of various ploidy and genome composition (RL, 2n = 26, and RRL or RLL, 3n = 39) (Ogielska et al. 2004, Zaleśna et al. 2011). Hybrids are sympatric with one of the parental species and live in mixed populations (called also genetic systems) composed of one of the parental species and hybrids (*P. lessonae*- *P. esculentus*, *P. ridibundus*- *P. esculentus*) or all-hybrid (*P. esculentus*- *P. esculentus*) composed of diploid and triploid individuals (Berger 1983, 1988, Graf and Polls-Pelaz 1989, Rybacki and Berger 2001, Plötner 2005). Reproduction and maintenance of the hybrid *P. esculentus* is the result of hybridogenesis, a unique way of hybrid reproduction (Schultz 1969). During this process, one of the parental sets of chromosomes of a hybrid (in this case R or L) is discarded from the germ line before meiosis and the other one is duplicated and clonally transmitted into gametes (Graf and Polls-Pelaz 1979). Some hybrids produce diploid gametes which give rise to triploid progeny (Uzzell et al. 1975, Rybacki 1994) and the combinations of genomes provided by the mother and by the father result in two types of triploids, namely RRL and RLL. Progeny of higher ploidy (4n and 5n) were also recorded but such individuals died before completion of metamorphosis or soon after (Christiansen 2009, Hermaniuk et al. 2013).

We applied cytogenetic techniques, *i.e.* silver, chromomycin A_3_ and Propidium Iodide (PI) staining, and fluorescent *in situ* hybridization with 28S rDNA probe commonly used in comparative studies of cold-blooded vertebrates: fish (Sola et al. 1992, Ráb et al. 1996, Boroń 1999, Rábová et al. 2001, 2003, Kirtiklis et al. 2010) and amphibians (Schmid 1982, Schmid et al. 1995). The aim of our study was to investigate the variability of NOR distribution patterns in metaphase chromosomes and interphase nuclei in water frogs, especially hybrid *P. esculentus* of various ploidy and genome composition. The results provide not only new data on distribution and/or polymorphism of NORs, but also may help to better understand inheritance of their patterns as the result of clonal genome transmission by hybrids during hybridogenetic gametogenesis.

## Materials and methods

### Animal sampling

In total, 407 individuals were analyzed: 272 adults and 135 newly metamorphosed juveniles. Juveniles were analyzed separately, as we expected a higher frequency of polyploids than in adults because tetraplois and pentaploids do not survive until adulthood (Hermaniuk et al. 2013, Christiansen 2009). Adult individuals (79 *P. lessonae*, 82 *P. ridibundus* and 111 *P. esculentus*) were collected from 11 natural populations in Central Europe (Table 1). Juveniles (15 *P. lessonae*, 52 *P. ridibundus* and 45 diploid, 18 triploid and 5 tetraploid *P. esculentus*) were obtained from *in vitro* artificial crosses. Their parents (*P. lessonae* LL, *P. ridibundus* RR, and *P. esculentus* RL, RRL, RLL) were collected from natural populations and served for 28 crosses resulting from various parental combinations (Table 2). Artificial crosses and rearing of progeny were done according to standard protocol for water frogs (Berger et al. 1994). Taxonomic identity of individuals was determined by morphology (Berger 1983, Plötner 2005, Kierzkowski et al. 2011) and at least one of the following methods: actinomycin D (AMD)-DAPI staining (Schweitzer 1976, Heppich et al. 1982, Ogielska et al. 2004), LDH electrophoresis and 17 microsatellite loci (both methods described in Hauswaldt et al. 2012).

Frogs were collected in years 2002–2009. All specimens used in this study were collected according to legal regulations concerning wild species protection under the following permits: Agency for Nature Conservation and Landscape Protection of the Czech Republic, Poodrí Protected Landscape Area, c.j. 0926/PO/2008/AOPK, permit S/0673/PO/2008/AOPK, and Polish Ministry of Environment Protection and Forestry for performing studies on protected species OP 4072/218 /96, OP 4072/218/98/4501, OP 4201/144/99, and II Local Commission for Ethics in Experiments on Animals 13/02.

**Table 1.** Number, collection site, sex, taxonomic status and genotype of adult individuals.

Population				Number of individuals									
nr	Name	type	Coordinates of sampling sites	lessonae		esculentus						ridibundus	
				LL		RL		RLL		RRL		RR	
				F	M	F	M	F	M	F	M	F	M
1	Baczysław	EE	53°49'53"N, 14°51'53"E							1			
2	Barycz River valley	RE	51°25'42"N, 16°56'49"E		1	11	43				4	23	54
			51°31'03"N, 17°02'04"E										
			51°31'05"N, 17°02'30"E										
			51°31'42"N, 17°49'54"E										
			51°34'23"N, 17°47'14"E										
3	Golczewo	EE	53°49'16"N, 14°58'10"E					1					
4	Horni Budlovice	LE	49°44'54"N, 18°26'30"E			2	1						
5	Mewia Rewa	LE	54°38'54"N, 18°27'57"E	1	8		1						
6	Piła	LE	53°01'30"N, 17°16'20"E	3	13								
7	Poznań	LE	52°03'40"N, 16°13'24"E	5									
8	Pruszowice	LE	51°11'01"N, 17°08'11"E			4	5						
9	Raków	LE	51°10'21"N, 17°16'36"E	12	17	8	5					3	2
10	Urwitałt	LE	53°48'19"N, 21°38'38"E	7	12								
11	Wysoka	EE	53°48'56"N, 14°50'53"E				1	2	8	8	6		
			53°48'53"N, 14°51'56"E										
			**Females/Males**	**28**	**51**	**25**	**56**	**3**	**8**	**9**	**10**	**26**	**56**
			**Total**	**79**		**81**		**11**		**19**		**82**	

### Chromosome preparation

Twenty-four hours before dissection, adults were injected intraperitoneally with 1 ml, and juveniles with 0.5 ml of 0.3% colchicine (Sigma- Aldrich, St. Louis, Mo., USA). Shortly before tissue preparation, the frogs were anesthetized with 0.25% water solution of 3-aminobenzoic acid ethyl ester (MS 222, Sigma-Aldrich). The intestine was dissected, hypotonized in distilled water (20 min for adults and 10 min for juveniles), and fixed in fresh-made fixative ethanol:glacial acetic acid (3:1) according to Heppich et al. (1982). Samples were stored in the fixative at –20°C until use. To obtain chromosome preparations, small pieces of the intestine epithelium were gently pressed against a slide to make tissue ‘prints’ that were immediately squashed under a coverslip in a drop of 70% acetic acid. The slides were placed on dry ice until frozen and then the coverslips were mechanically removed. The squashes were air-dried overnight and stored at –20°C. A minimum of 10 metaphases were analyzed for each individual.

Chromosome preparations of three *P. esculentus* individuals were made in a different way. The animals were injected intraperitoneally with 0.1% colchicine (10µl/g body weight) 2.5 h before dissection, femur bones were removed from euthanized frogs, epiphyses were clipped off, and bone marrow cavities were immediately flushed with 0.075 M KCl solution applied with a syringe at 37°C. Bone marrow tissue was pressed through a small-mesh gauze, flushed out with 0.075 M KCl solution, then placed in a centrifuge tube filled up to 7 ml with 0.075 M KCl solution, and incubated at 37°C for 20 min. Hypotonic treatment was stopped by fixation in absolute methanol:glacial acetic acid (3:1), and cell suspension was centrifuged at 1,500 rpm for 10 min. The supernatant containing fat droplets was discarded with a Pasteur pipette and fresh fixative was added up to 5 ml. The pellet was re-suspended by agitation and kept in a freezer for 20 min, then centrifuged again at 1,500 rpm for 10 min. The procedure (centrifugation, fixative exchange, and cooling) was repeated 3 times. The suspension was then transferred to a 1-ml syringe, dropped onto slides (previously cleaned in HCl:ethanol, 3:1), and finally air-dried. Chromosome preparations obtained with either protocol were suitable for all staining methods used in this study. However, chromosomes prepared from bone marrow cells were more uniformly condensed and thus more suitable for FISH than the chromosomes obtained from gut epithelium prints that varied in the degree of condensation (Zaleśna et al. 2011).

### Chromosome banding

The nucleolus organizer regions (NORs) were stained in all individuals by the silver nitrate technique (Ag-NOR). Chromosome slides of 15 randomly selected individuals were sequentially stained with chromomycin A**_3_** (CMA_3_) and AgNO_3_. All individuals that displayed another number of NORs than one per a haploid set after Ag-NOR staining were examined by PI or CMA_3_. For 22 individual (9 RR, 3 LL, 5RL, 1 RRL, 3 LLR, 1 RRLL) we applied the FISH method with 28S rDNA as a probe.

*Silver staining.* We followed the protocol of Howell and Black (1980). A few drops of freshly prepared silver nitrate buffer (0.5g AgNO_3_/1ml H_2_O/0.5ml gelatin solution, *i.e.*1g gelatin/0.5ml formic acid/50ml bi-distilled water) were applied to each preparation. Slides were covered with a nylon mesh and incubated in a humid chamber at 60°C for 1min, washed in distilled water and air-dried. If the chromosomes were poorly visible, they were counterstained with DAPI. The number of active rDNA loci was documented by simultaneous use of fluorescence and incandescent light.

*Chromomycin A_3_ (CMA_3_).* The method was used according to Schweitzer (1976). Slides were incubated in McIlvain buffer pH 7.0 with 2.5mM MgCl _2_ for 10 min and stained with CMA**_3_** solution (0.5 mg/mL buffer and 2.5mM MgCl_2_) for 15 min in the dark, briefly rinsed in buffer and counterstained with methyl green for 15 min (0.175g methyl green/50ml buffer). After washing in buffer, slides were stained with DAPI solution (0.5µg/ml) for 10 min in the dark and briefly rinsed with buffer. Tissue prints were mounted in 50% glycerol and analyzed using fluorescence illumination. After CMA_3_ staining, preparations were faded and cleaned in xylene and benzene, each bath for 2 min, and then stained with AgN0_3_.

*Propidium Iodode staining after denaturation of chromosomes (PI).* Tissue prints were dehydrated in 70%, 85% and 95% ethanol (30 sec each wash), air-dried at room temperature and denatured in 70% deionized formamide in 2xSSC at 70°C for 3.5 min. Slides were immediately dehydrated in chilled 70% ethanol from a freezer for 2 min, then in 85% and 95% at room temperature for 30 sec. Chromosomes were stained with PI (200ng/ml).

### Extraction and labeling of 28S rDNA

We applied the FISH method with 28S rDNA as a probe, according to the protocol of Traut et al. (1999). Isolation of 28S rDNA was carried out with use of commercial GeneMATRIX Bio-Trace Purification Kit (Eurix). DNA amplification was performed using PCR reaction with suitable mixture: 2.5µl PCR buffer in 15 mM MgCl2 (Eurix), 1µl 5mM dNTP (Eurix), 1µl 10mM primer A (5’-TCC GTG TTT CAA GAC GGG - 3’) and 1 µl 10mM primer B (3’-ACC CGC TGA ATT TAA GCA T -5’), 1µl 1U/µl Polymerase (OptiTaq-Eurix), 1µl matrix DNA and 17.5µl water. PCR conditions included initial denaturation in 94°C for 5 min, followed by 35 cycles: 30 s denaturation (94°C), 30 s annealing (58°C), 1 min elongation (72°C), and 5 min of final elongation (72°C). The 28S rDNA probe was labelled with tetramethyl-rhodamine-6-dUTP (Roche, 11093088910) using a Nick Translation Mix according to the manufacturer’s protocol (Roche, 10976776001). Then, DNA was precipitated by ethanol for purification and concentrated by adding one-tenth volume of 3M sodium acetate and 2.5 volume of chilled 96% ethanol from a freezer. The mixture was incubated for at least 15 min at –80°C. The precipitated DNA was spun at 15,000 rpm for 15 min at 4°C and the supernatant was discarded. The pellet was washed with 50–100 ml of chilled 70% ethanol and spun at 15,000 rpm for 10 min at 4°C. DNA was dried at 37°C for 15–20 min.

### Fluorescent in situ hybridization (FISH)

Slides were incubated in 100 µg/ml DNase-free RNase A in 2x SSC for 1 h at 37° C in a humid chamber and then washed twice in 2xSSC at room temperature for 5 min, dehydrated in ethanol series (70%, 85%, and 95%, 30 s each) and air-dried. Chromosome preparations were denatured in 70% deionized formamide, 2x SSC at 70° C for 3.5 min, dehydrated immediately for 2 min in ice-cold 70% ethanol, then in 85% and 95% ethanol for 30s at room temperature, and finally air-dried. The 28S rDNA probe was dissolved in hybridization mixture consisted of 100% deionized formamide and 20% dextran sulfate in proportion 1:1. The mixture was denatured at 90° C for 5 min and then immediately placed on ice for 3 min. 20 µl of the probe was applied to each slide and covered with a coverslip. Hybridization lasted overnight in a dark humid chamber at 37° C. After hybridization, the coverslip was removed by rinsing with 50% formamide in 2xSSC at 42°C twice for 7 min and the slide was washed 3 times (7 min each) in 1xSSC at 42°C and then in 2xSSC at room temperature for 30 s. Chromosomes were counterstained with DAPI in Vectashield antifade buffer (Cambio, Cambridge, UK).

### Image processing

Chromosomes were analyzed in Olympus Provis AX 70 or Carl Zeiss Axioskop 20 microscopes equipped with fluorescence lamp HBO50 and appropriate filters. Images were recorded with Olympus DP30BW CCD and cooled Carl Zeiss AxioCam HRc CCD cameras and processed using AxioVision and Lucia ver. 2.0 (Laboratory Imaging) softwares.

## Results

**Figure 1.**
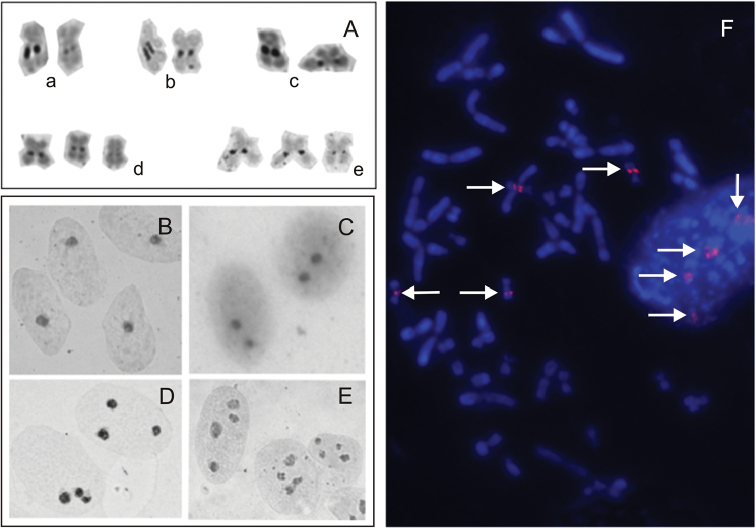
**A** The variability of size of AgNORs (black bands) on chromosomes 10 **a** *Pelophylax ridibundus* **b** *P. lessonae* **c** *P. esculentus* RL **d** *P. esculentus* RRL **e** *P. esculentus* LLR **B–E** Interphase nuclei of *P. esculentus* with Ag-nucleoli visualized as black dots by AgNO_3_ **B** diploid RL with 1 AgNOR **C** diploid RL with 2 AgNORs **D** triploid RRL with 3 AgNORs **E** triploid RRL with 4 AgNORs **F** metaphase chromosomes and interphase nuclei of the same tetraploid P. esculentus RRLL with 4 sites of hybridization with 28S rDNA probe (FISH) (arrows).

The number of NORs was visualized for all (407) studied individuals (see Tables 1 and 2), *i.e.* 354 diploids (242 adults and 112 juveniles RR, LL, RL), 48 triploids (30 adults and 18 juveniles RRL and LLR), and 5 tetraploids RRLL. The results are summarized in Table 3. After AgNO_3_ staining, one NOR per a haploid set was identified in secondary constrictions on long arms of chromosomes 10 in 341 individuals (83.8%), and in these individuals the number of NORs reflected the ploidy level. Two NORs were detected in 302 (85.3%) diploid individuals belonging to all studied taxa (232, *i.e.* 96% adults and 70, *i.e*. 62.5% juveniles), regardless of their capture site and taxonomic status. In all analyzed metaphases of the species (*P. lessonae* and *P. ridibundus*) and hybrids (*P. esculentus* 2n and 3n) , NORs exhibited variations in size after AgNO_3_ and CMA_3_ stainings, bands at one of the homologs were slightly thicker than at the other one (Fig. 1A). The number of Ag-NORs visible on metaphase plates was the same as the number of AgNO_3_ stained nucleoli in interphase nuclei of the same individual (Fig. 1B–F). Three NORs were detected in 34 (70.8%) triploid hybrids, both RRL and RLL (26, *i.e*. 86.7% adults and 8, *i.e*. 44.4% juveniles), and four Ag-NORs were recorded in all metaphase plates of five tetraploids (RRLL).

**Figure 2.**
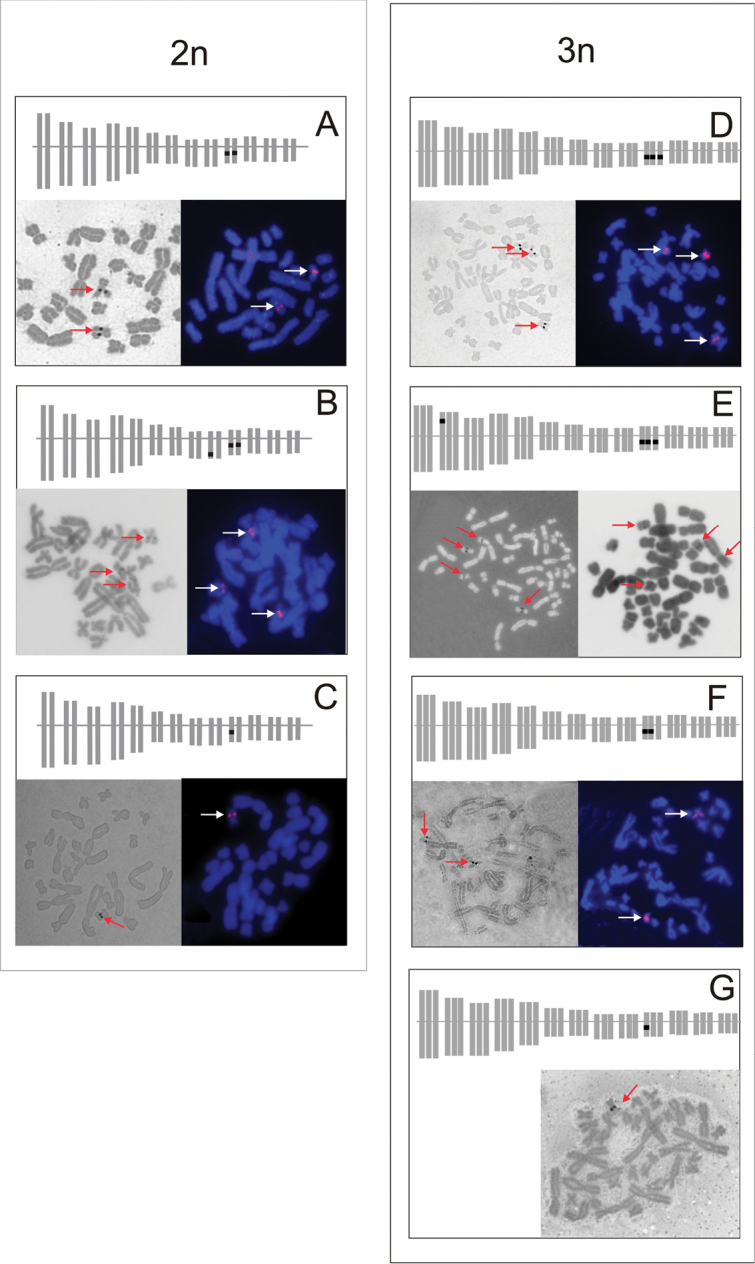
Localization and number of NORs (arrows) in diploid and triploid water frogs. Left column represent diploids (**A–C**) and right column represent triploids (**D–G**). Each picture is composed of a diagram of karyotype with black dots representing NORs and metaphase plates stained with silver (AgNOR), chromamycin A_3_ (CMA_3_), propidium iodide after denaturation (DPI) or after fluorescent *in situ* hybridization with 28S rDNA probe (FISH). **A** *P. lessonae* LL with 2 NORs, AgNORs (left) and FISH (right) **B** *P. esculentus* RL with 4 NORs on chromosome 9, CMA_3_ (left) and FISH (right) **C** *P. esculentus* RL with 1 NOR, AgNORs (left) and FISH (right) **D** *P. esculentus* LLR with 3 NORs, AgNORs (left) and FISH (right) **E** *P. esculentus* RRL with 4 NORs, AgNOR (left) and DPI (right) **F** *P. esculentus* RRL with 2 NORs, AgNORs (left) and FISH (right) **G** *P. esculentus* RRL with 1 NOR, CMA_3_.

Results obtained by silver staining corresponded well with those stained with CMA_3_, PI or FISH (Fig. 2A–G). Only one individual *P. ridibundus* displayed one active NOR after AgNO_3_ although two GC-rich chromatin blocks located in both chromosomes 10 were visulized after PI and CMA_3_. All other individuals displayed the same number of PI and CMA_3_ positive sites or 28S rDNA hybridizing signals as visualized by silver staining.

**Table 2.** Number, sex, taxonomic status and genotype of juvenile progeny of artificial crosses.

	Number of individuals											
	*lessonae*		*esculentus*								*ridibundus*	
	LL		RL		RRL		RLL		RRLL		RR	
	F	M	F	M	F	M	F	M	F	M	F	M
	7	8	32	13	14	1	1	2	4	1	44	8
**Total**	**15**		**45**		**15**		**3**		**5**		**52**	

**Table 3.** Number of NORs in individuals of various ploidy level. The number of NORs that is in agreement with ploidy level is in bold.

Ploidy and genome composition	Number of individuals (adults and juveniles)				
		Number of NORs			
		1	2	3	4
2n (LL, RR, RL)	354	51	**302**	1	
3n (RRL, LLR)	48	2	3	**34**	9
4n (RRLL)	5				**5**
Total	407				

### NOR polymorphism

In 66 individuals (16.2%, 13 adult and 53 juveniles), the number of NORs differed from the expected values. Lack of NORs was relatively more frequent (13.8%, 56 out of 407 individuals) than extra NORs (2.46%, 10 out of 407 individuals), regardless of genomic compositions (RR, LL, RRL and LLR) and origin of individuals. Eight diploid adult individuals (3 diploid *P. esculentus*, 3 *P. lessonae* and 2 *P. ridibundus*) and 43 diploid juveniles (11 *P. esculentus*, 6 *P. lessonae*, and 26 *P. ridibundus*) had NORs only on one chromosome of pair 10 (Fig. 2C). Two triploid adults (one LLR and one RRL) and one juvenile displayed two, instead of three NORs (Fig. 2F). Two other triploid RRL individuals (1 adult and 1 juvenile) displayed one, instead of expected three NORs in chromosomes 10 (Fig. 2G).

Additional NORs were detected in ten individuals. The extra NORs were located within additional secondary constrictions on one of the homologs of chromosomes other than 10. Extra NORs were found in one adult *P. ridibundus* in the distal position of the long arm of chromosome 9 (Fig. 2 B). In interphase nuclei of this individual we found the corresponding number of three Ag-nucleoli (Fig. 1D). Extra NORs were also observed on short arms of chromosomes 2 in nine triploid *P. esculentus* RRL: one adult and eight juveniles (Fig. 1E).

### Inheritance of NOR patterns

**Figure 3.**
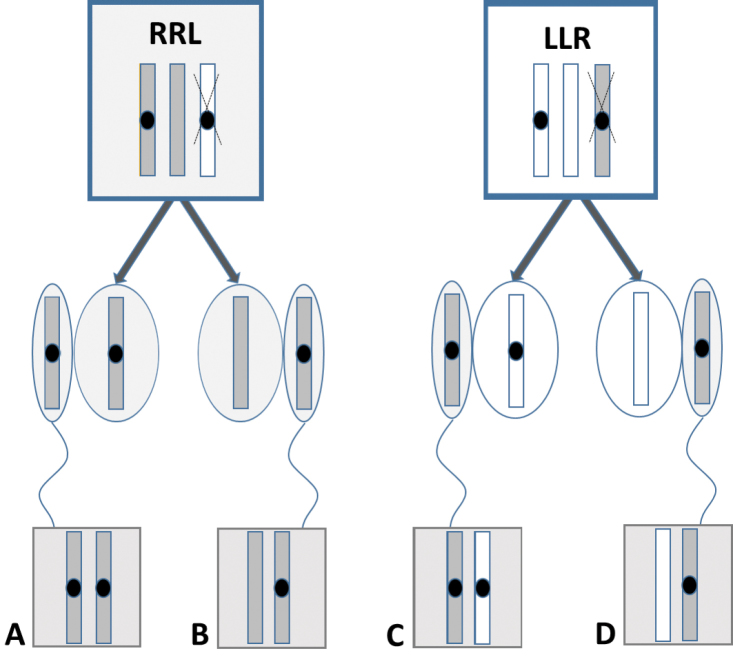
Inheritance of NORs by progeny of triploid females RRL (right) and RLL (left). The chromosome set represented by one copy is eliminated before oogenesis (marked by X) and the double one is segregated into eggs (represented by ovals). After fertilization (in this case by *ridibundus* sperm), two types of progeny arises: with two (**A** and **C**) and with one (**B** and **D**) NOR. The lack of NORs are transmitted either by *lessonae* (white) or by *ridibundus* (dark grey) chromosome sets. NORs are represented by black dots.

The lack of one NOR was also detected in diploid progeny of two triploid RRL females and one LLR female. After AMD/DAPI staining we discovered that in the case of the RRL females the lack of NORs was inherited together with the haploid set of the *ridibundus* chromosomes, whereas in the case of the LLR female it was inherited with the haploid set of the *lessonae* chromosomes. Among diploid progeny of these females (altogether 37 juveniles) we observed 16 individuals with two NORs and 21 individuals with only one NOR. Each individual was stained with AgNOR and seven of them were confirmed by FISH. Thus, despite that the genomes of ova were the same (R in the case of RRL and L in the case of LLR females), the females produced in fact two types of gametes – with and without NORs (Fig. 3).

## Discussion

The majority of individuals displayed one NOR per a haploid set of chromosomes (n = 13) located within the secondary constriction on the long arm of chromosome 10 (named 9 in Biriuk et al. 2015). This result is in accordance with other reports on water frogs (Koref-Santibanez and Günther 1980, Schmid 1982, Heppich et al. 1982, Vitelli et al. 1982, Spasič-Bošković et al. 1999, Ogielska et al 2004, Martirosyan and Stepanyan 2009, Zaleśna et al. 2011). Localization of NORs on chromosomes 10 is a conservative trait in the ranid frogs (Birstein 1984).

Ag-NORs observed in metaphase chromosomes in water frogs were also recognized in interphase nuclei, as was reported by Schmid (1982) and Biriuk et al (2015). Similar results were also observed in the lungless salamander *Onychodaxtylus fischeri* (Iizuka et al 2013), caecilians *Ichthyophis*, *Uraeotyphlus* and *Gegeneophis* (Venu 2014), and the frog *Physalaemus petersi* (Lourenço et al. 1998).

The number of visualized NORs corresponded well with the ploidy level, as expected for individuals with only one pair of NOR-bearing chromosomes. Thus, diploids had two NORs, triploids - three NORs, and tetraploid - four NORs. However, number of NORs in interphase nuclei may be misleading in specific cases. As we demonstrated in this study, interphase nuclei in a diploid individual with an extra NOR on chromosome 9 displayed the same pattern as in a triploid individual with three NORs on the homologous chromosomes 10.

### Polymorphism of NORs

The variability of AgNOR sizes in individuals from the same populations may reflect different amount of rDNA (Miller and Brown 1969 for *Bufo marinus* and Macgreor et al. 1977 for *Plethodon cinereus*). Various sizes of AgNORs in frogs were described by Schempp and Schmid (1981). Schmid (1982) found a correlation between sizes of bands in secondary constrictions of AgNO_3_ stained chromosomes and the intensity of CMA_3_ signals which were proportional to the amount of rDNA in NORs. We also found variability of NOR sizes in chromosomes 10, however Biriuk et al. (2015) reported that the differences measured as a relative length were not significant in 65 investigated individuals of diploid and triploid green frogs. The polymorphism of NORs can also reflect geographical karyotypic variations (Amaro et al. 2012, Silva et al. 2000, Quinderé et al. 2009, Bi et al. 2009). However, we found no variations of NOR number and size in water frogs deriving from different populations.

Extra NORs were also reported in *Rana catesbeiana* (*Lithobates catesbeianus*) that displayed from two to seven Ag-positive small NORs per haploid karyotype, apart from one “standard” NOR on the chromosome 10 (Schmid 1978a,b). In the same species, Vitelli et al. (1982) revealed the standard NORs on the chromosomes 10 by FISH with 18S+28S rDNA probe, whereas 5S rDNA sequences were clustered near the centromere on the short arm of chromosome 12 and corresponded to small Ag-positive bands. Other small NORs were not confirmed neither by FISH with the 18S + 28S rDNA nor by 5S rDNA probes. FISH with 18S+28S rDNA probe in *Pelophylax esculentus* (Vitelli et al. 1982) displayed signals in chromosome 10, and with 5S rDNA probe at telomere of short arm of chromosome 5. Moreover, FISH with 28S rDNA probe, as well as CMA_3_ and PI stainings revealed that extra NORs corresponded with the extra Ag-positive signals on the chromosomes 9. Neither of these locations corresponded to extra NORs that were observed in our study.

More common was the lack of NORs (13.8% of all animal studied herein). We observed a lack of one NOR (2n and 3n) or two NORs (only in 3n), both in metaphase plates and interphase nuclei. Lack of NORs in amphibians was also reported by Schimd (1982) in *Bombina variegata*, *Xenopus laevis*, and *Bufo fowleri*. According to Schmid (1982) and Motovali-Bashi et al. (2004), the causes of deletion or amplification of NORs were usually unequal meiotic divisions, sister chromatid exchanges or disruption of DNA replication.

Intraspecific polymorphism of the location of extra NORs was also reported in *Hyla chrysoscelis* and *H. versicolor* (Wiley et al. 1989, Wiley and Little 2000), *H. nana* (Medeiros et al. 2003), *Bufo terrestris* (Foote et al. 1991), *Agalychnis callidryas* (Schmid et al. 1995), *Physalaemus petersi* (Lourenço et al. 1998), *P. cuvieri* (Quinderé et al. 2009), *Phyllomedus arhodei* and *P. nordestina* (Barth et al. 2013). Possible mechanisms of the dispersion of NOR sites were translocations involving chromosomal segments containing NORs, however such chromosome rearrangements were connected with change in morphology of the NOR chromosomes (Lourenço et al. 1998). Other possible mechanisms involved in dispersion of NORs in anuran genomes could be inversions, transpositions by mobile genetic elements containing NORs (Wiley et al. 1989, Foote et al. 1991, Lourenço et al. 1998, Schmid et al. 1995, King et al. 1990, Bruschi et al. 2012, Carvalho et al. 2014) or rDNA amplification (Barth et al. 2013). However, in the case of water frogs presented here, translocations involving chromosomal segments containing NORs on chromosomes 2 and 9 seems unlikely because chromosomes with extra NORs did not vary in length and shape from relative chromosomes in other individuals.

### Inheritance of NOR patterns

The lack of one NOR was detected in both RRL and LLR triploid females and was associated with the *ridibundus* and *lessonae* chromosome sets, respectively. Triploid water frogs produce haploid gametes and transmit the chromosome set that is represented by two copies (R in RRL and L in LLR) (Christiansen 2009). Because one of the doubled sets lacked NOR, we were able to trace this character and we found it in about a half of progeny whereas the other half inherited NORs (Fig. 3). This intriguing result deserves more attention and further studies because it may serve as an indicator of clonal versus recombined inheritance of the doubled chromosome sets, as was possible in triploid green toads *Bufo baturae* (Stöck et al. 2002, 2012). This all-triploid population is represented by males and females that carry NORs in two chromosome sets whereas the third one lacks NORs. This character, known also from other amphibian species (Schmid 1982, Motovali-Bashii et al. 2004, this study), was probably conserved in an isolated population in the Karakoram Mountains in Pakistan. Triploid *Bufo baturae* females produce diploid ova containing one (recombined) set with NORs whereas the set without NOR is transmitted without recombination (clonally).

Finally, we conclude that polymorphism concerning the number and localization of NORs in water frogs was characteristic of both *lessonae* and *ridibundus* genomes, and – as expected – was observed in individuals regardless of their taxonomic position (*P. lessonae*, *P. ridibundus* and *P. esculentus*), ploidy level (2n, 3n, 4n), genomic constitution (RR, LL, RRL, RLL and LLRR), and collection site. The number of active silver-stained NORs reflected ploidy levels: two in diploids, three in triploids, and four in tetraploids, and therefore we believe that there is no diploidization of polyploids in water frogs, as has been observed in some of polyploid amphibians (for review see Schmid et al. 2015). The variability of NORs may be hereditary, what is best represented by inheritance of lack of NORs by the progeny of triploid females.

## Acknowledgements

The study was financially supported by grants 3490/B/P01/2007/33 and 3946/B/P01/2009/36 provided by the Polish Ministry of Science and Higher Education. We are grateful to Katarzyna Lipiec-Sidor for technical help with figures arrangement and setup.

## References

[B1] Amaro RC Rodrigues MT Yonenaga-Yassuda Y Carnaval AC (2012) Demographic processes in the montane Atlantic rainforest: Molecular and cytogenetic evidence from the endemic frog *Proceratophrys boiei*. Molecular Phylogenetics and Evolution 62: 880–888. https://doi.org/10.1016/j.ympev.2011.11.004 22108674 10.1016/j.ympev.2011.11.004

[B2] Barth A Souza VA Solé M and Costa MA (2013) Molecular cytogenetics of nucleolar organizer regions in *Phyllomedsua* and *Phasmahyla* species (Hylidae. Phyllomedusinae): a cytotaxonomic contribution. Genetics and Molecular Research 12(3): 2400–2408. https://doi.org/10.4238/2013.July.15.323979880

[B3] Berger L (1983) Western Palearctic water frogs (Amphibia, Ranidae), Systematics, genetics and population compositions. Experiential 39: 127–130. https://doi.org/10.1007/BF01958859

[B4] Berger L (1988) On the origin of genetic systems in European water frog hybrids. Zoologica Poloniae 35: 5–32.

[B5] Berger L Rybacki M Hotz H (1994) Artificial fertilization of water frogs. Amphibia-Reptilia 15: 408–413. https://doi.org/10.1163/156853894X00452

[B6] Bi K Bogart JP Fu J (2009) A populational survey of 45S rDNA polymorphism in the Jefferson Salamander using FISH. Current Zoology 55: 145–149.

[B7] Biriuk OV Rosanov JM Litvinchuk SN Pasynkova RA (2015) Variability of nucleolus organizer region sizes in green frogs karyotypes. The Journal of V.N. Karazin Kharkiv National University Series Biology 25: 145–155.

[B8] Birstein VJ (1984) Localization of NORs in karyotypes of four *Rana* species. Genetica 64: 149–154. https://doi.org/10.1007/BF00115338

[B9] Boroń A (1999) Banded karyotype of spined loach *Cobitis taenia* and triploid Cobitis from Poland. Genetica 3: 293–300. https://doi.org/10.1023/A:1003939813878 10.1023/a:1003939813878 10761112

[B10] Bruschi DP Busin CS Siqueira S Recco-Pimentel SM (2012) Cytogenetic analysis of two species in the *Phyllomedusa hypochondrialis* group (Anura, Hylidae). Hereditas 149: 34–40. https://doi.org/10.1111/j.1601-5223.2010.02236.x 22458439 10.1111/j.1601-5223.2010.02236.x

[B11] Carvalho MA Rodrigues MT Siqueira S Garcia C (2014) Dynamics of chromosomal evolution in the genus *Hypsiboas* (Anura: Hylidae). Genetics and Molecular Research 13(3): 7826–7838. https://doi.org/10.4238/2014.September.26.21 25299097 10.4238/2014.September.26.21

[B12] Christiansen DG (2009) Gamete types, sex determination and stable equilibria of all-hybrid populations of diploid and triploid edible frogs (*Pelophylax esculentus*). BMC Evolutionary Biology 9: 1–16. https://doi.org/10.1186/1471-2148-9-135 19527499 10.1186/1471-2148-9-135 PMC2709657

[B13] Foote DL Wiley JE Little ML (1991) Ribosomal RNA gene site polymorphism in *Bufo terrestris*. Cytogenet Cell Genet 57(4): 196–9. https://doi.org/10.1159/000133145 1743073 10.1159/000133145

[B14] Graf JD Polls-Pelaz M (1989) Evolutionary genetics of the *Rana esculenta* complex. In: Dawley RM, Bogart JP (Eds) Evolution and Ecology of Unisexual Vertebrates The New York State Museum, Albany, 289–302.

[B15] Hauswaldt SJ Höer M Ogielska M Christiansen DG Dziewulska-Szwajkowska D Czernicka E Vences M (2012) A simplified molecular method for distinguishing among species and ploidy levels in European water frogs (*Pelophylax*). Molecular Ecology Resources 12(5): 797–805. https://doi.org/10.1111/j.1755-0998.2012.03160.x 22716763 10.1111/j.1755-0998.2012.03160.x

[B16] Heppich S Tunner HG Greilhuber J (1982) Premeiotic chromosome doubling after genome elimination during spermatogenesis of the species hybrid *Rana esculenta*. Theoretical and Applied Genetics 61: 101–104. https://doi.org/10.1007/BF00273874 24270328 10.1007/BF00273874

[B17] Hermaniuk A Pruvost NBM Kierzkowski P Ogielska M (2013) Genetic and cytogenetic characteristics of pentaploidy in water frogs. Herpetologica 69: 36–45. https://doi.org/10.1655/HERPETOLOGICA-D-12-00037

[B18] Howell WM Black DA (1980) Controlled silver-staining of nucleolus organizer regions with a protective colloidal developer: a 1-step method. Experientia 36: 1014–1015. https://doi.org/10.1007/BF01953855 6160049 10.1007/BF01953855

[B19] Iizuka K Matsuda Y Yamada T Nakazato T Sessions SK (2013) Chromosomal localization of the 18S and 28S ribosomal RNA genes using FISH and AgNO_3_ banding in *Hynobius quelpaertensis*, *H. tsuensis* and *Onychodactylus koreanus* (Urodela: Hynobiidae). Current Herpetology 32(2): 89–101. https://doi.org/10.5358/hsj.32.89

[B20] Kierzkowski P Paśko Ł Rybacki M Socha M Ogielska M (2011) Genome dosage effect and hybrid morphology - the case of the hybridogenetic water frogs of the *Pelophylax esculentus* complex. Annales Zoologici Fennici 48: 56–66. https://doi.org/10.5735/086.048.0106

[B21] King M Contreras N Honeycutt RL (1990) Variation within and between nucleolar organizer regions in Australian hylid frogs (Anura) shown by 18S 1 28S *in situ* hybridization. Genetica 80: 17–29. https://doi.org/10.1007/BF00120116 2323563 10.1007/BF00120116

[B22] Kirtiklis L Porycka K Boroń A Coutanceau J-P Dettai A (2010) Use of the Chromosomal Co-location of the Minor 5S and the Major 28S rDNA as a Cytogenetic Marker within the Genus *Leuciscus* (Pisces, Cyprinidae). Folia biologica (Kraków) 58: 245–249. https://doi.org/10.3409/fb58_3-4.245-249 10.3409/fb58_3-4.245-249 20968192

[B23] Koref-Santibanez S Günther R (1980) Karyological and serological studies in *Rana lessonae*, *R. ridibunda* and their hybrid “*R. esculenta*” (Amphibia, Anura). Genetica 52/53: 195–207. https://doi.org/10.1007/BF00121828

[B24] Lourenço LB Recco-Pimentel SM Cardoso AJ (1998) Polymorphism of the nucleolus organizer regions (NORs) in *Physalaemus petersi* (Amphibia, Anura, Leptodactylidae) detected by silver staining and fluorescence in situ hybridization. Chromosome Research 6: 621–628. https://doi.org/10.1023/A:1009253410553 10099875 10.1023/a:1009253410553

[B25] Macgreor HC Vlad M Barnett L (1977) An investigation of some problems concerning nucleolus organizers in salamanders. Chromosoma 59(4): 283–299. https://doi.org/10.1007/BF00327970 837805 10.1007/BF00327970

[B26] Martirosyan A Stepanyan I (2009) Features of the karyotypes of *Pelophylax ridibundus* Pallas, 1771 and *Rana macrocnemis* Boulenger, 1885 (Amphibia: Ranidae) from Armenia. Comparative Cytogenetics 3(1): 11–24. https://doi.org/10.3897/compcytogen.v3i1.4

[B27] Medeiros LR Rossa-Feres DC Recco-Pimentel SM (2003) Chromosomal differentiation of *Hyla nana* and *Hyla sanborni* (Anura, Hylidae) with a description of NOR polymorphism in *H. nana*. Journal of Heredity 94(2): 149–154. https://doi.org/10.1093/jhered/esg019 12721226 10.1093/jhered/esg019

[B28] Miller L Brown DD (1969) Variation in the activity of nucleolar organizers and their ribosomal gene content. Chromosoma 28: 430–444. https://doi.org/10.1007/BF00284259 5364411 10.1007/BF00284259

[B29] Motovali-Bashii M Hojatia Z Walmsleyb RM (2004) Unequal sister chromatid exchange in the rDNA array of *Saccharomyces cerevisiae*. Mutation Research 564: 129–137. https://doi.org/10.1016/j.mrgentox.2004.08.004 15507377 10.1016/j.mrgentox.2004.08.004

[B30] Ogielska M Kierzkowski P Rybacki M (2004) DNA content and genome composition of diploid and triploid water frogs belonging to the *Rana esculenta* complex (Amphibia, Anura). Canadian Journal of Zoology 82: 1894–1901. https://doi.org/10.1139/z04-188

[B31] Plötner J (2005) Die westpaläarktischen Wasserfrösche – Von Märtyrern der Wissenschaft zur biologischen Sensation. Laurenti Verlag, Bielefeld, 160 pp.

[B32] Quinderé YRSD Lourenço LB Andrade GV Tomatis C Baldo D Recco-Pimentel SM (2009) Polytypic and polymorphic cytogenetic variations in the widespread anuran *Physalaemus cuvieri* (Anura, Leiuperidae) with emphasis on nucleolar organizing regions. Biological Research 42: 79–92. https://doi.org/10.4067/s0716-97602009000100008 19621135

[B33] Ráb P Reed KM Ponce de Leon FA Phillips RB (1996) A new method for detecting nucleolus organizer regions in fish chromosomes using denaturation and propidium iodide staining. Biotechnic and Histochemistry 71(3): 157–162. https://doi.org/10.3109/10520299609117153 8724442 10.3109/10520299609117153

[B35] Rábová M Ráb P Ozouf-Costaz C (2001) Extensive polymorphism and chromosomal characteristics of ribosomal DNA in a loach fish, *Cobitis vardarensis* (Ostariophysi, Cobitidae) detected by different banding techniques and fluorescence in situ hybridization (FISH). Genetica 111(1–3): 413–22. https://doi.org/10.1023/A:1013763903513 11841184 10.1023/a:1013763903513

[B34] Rábová M Ráb P Ozouf-Costaz C Ene C Wanzeböck J (2003) Comparative cytogenetics and chromosomal characteristics of ribosomal DNA in the fish genus Vimba (Cyprinidae). Genetica 118(1): 83–91. https://doi.org/10.1023/A:1022965302614 12733667 10.1023/a:1022965302614

[B36] Reeder RH (1990) rRNA synthesis in the nucleolus. Trends in Genetics 6: 390–395. https://doi.org/10.1016/0168-9525(90)90298-K 2087780 10.1016/0168-9525(90)90298-k

[B37] Rybacki M (1994) Diploid males of *Rana esculenta* from natural populations in Poland producing diploid spermatozoa. Zoologica Poloniae 39: 518–518.

[B38] Rybacki M Berger L (2001) Types of water frog populations (*Rana esculenta* complex) in Poland. Mitteilungen aus dem Museum für Naturkunde in Berlin (Zoologische Reihe) 77: 51–57. https://doi.org/10.1002/mmnz.20010770109

[B39] Schempp W Schmid M (1981) Chomosome banding in Amphibia. VI. BrdU replication patterns in Anura and demonstration of XX/XY chromosomes in *Rana esculenta*. Chromosoma 83: 697–710. https://doi.org/10.1007/BF00328528 6975199 10.1007/BF00328528

[B40] Schmid M (1978a) Chromosome banding in Amphibia. I. Constitutive heterochromatin and nucleolus organizer regions in *Bufo* and *Hyla*. Chromosoma 66: 361–388. https://doi.org/10.1007/BF00328536

[B41] Schmid M (1978b) Chromosome banding in Amphibia. II. Constitutive heterochromatin and nucleolus organizer regions in Ranidae, Microhylidae and Rhacophoridae. Chromosoma 68: 131–148. https://doi.org/10.1007/BF00287145

[B42] Schmid M (1982) Chromosome Banding in Amphibia VII. Analysis of the Structure and Variability of NORs in Anura Chromosoma 87: 327–344.

[B43] Schmid M Feichtinger W Weimer R Mais C Bolaños F Leon P (1995) Chromosome banding in Amphibia. XXI. Inversion polymorphism and nucleolus organizer regions in *Agalychnis callidryas* (Anura, Hylidae). Cytogenetetics and Cell Genetics 69: 8–26. https://doi.org/10.1159/000431388 10.1159/000133929 7835080

[B44] Schmid M Evans BJ Bogart JP (2015) Polyplody in Amphibia. Cytogenetic and Genome Research 145: 315–330. 26112701 10.1159/000431388

[B45] Schultz RJ (1969) Hybridization, unisexuality, and polyploidy in the teleost *Poeciliopsis* (Poeciliidae) and other vertebrates. The American Naturalist 103: 605–619. https://doi.org/10.1086/282629

[B46] Schweitzer D (1976) Reverse fluorescent chromosome banding with chromomycin and DAPI Chromosoma 58: 307–324. 10.1007/BF00292840 137107

[B47] Silva APZ Junior FAB Haddad CFB Kasahara S (2000) Karyotypes and nucleolus organizer regions in four species of the genus *Physalaemus* (Anura, Leptodactylidae). Iheringia, Série Zoologia 88: 159–164.

[B48] Sola L Rossi AR Iaselli V Rasch EM Monaco PJ (1992) Cytogenetics of bisexual/unisexual species of *Poecilia*. II. Analysis of heterochromatin and nucleolar organizer regions in *Poecilia mexicana mexicana* by C-banding and DAPI, quinacrine, chromomycin A_3_, and silver staining. Cytogenetics and Cell Genetics 60(3–4): 229–35. https://doi.org/10.1159/000133346 1380417 10.1159/000133346

[B49] Spasič-Bošković O Krizmanić I Vujošević M (1999) Population composition and genetic variation of water frogs (Anura: Ranidae) from Yugoslavia. Caryologia 52: 9–20. https://doi.org/10.1080/00087114.1998.10589148

[B50] Stöck M Lamatsch DK Steinlein C Epplen JT Grosse WR Hock R Klapperstück T Lampert KP Scheer U Schmid M Schartl M (2002) A bisexually reproducing all-triploid vertebrate. Nature Genetics 30: 325–328. https://doi.org/10.1038/ng839 11836500 10.1038/ng839

[B51] Stöck M Ustinova J Betto-Colliard C Schartl M Moritz C Perrin N (2012) Simultaneous Mendelian and clonal genome transmission in a sexually reproducing, all-triploid vertebrate. Proceedings of the Royal Society B Biological Sciences 279: 1293–1299. https://doi.org/10.1098/rspb.2011.1738 21993502 10.1098/rspb.2011.1738 PMC3282369

[B52] Traut W Sahara K Otto TD Mařec F (1999) Molecular differentiation of sex chromosomes probed by comparative genomic hybridization. Chromosoma 108: 173–180. https://doi.org/10.1007/s004120050366 10398846 10.1007/s004120050366

[B53] Uzzell T Berger L and Günther R (1975) Diploid and triploid progeny from a diploid female of *Rana esculenta* (Amphibia, Salientia). Proceedings of the Academy of Natural Sciences of Philadelphia 127: 81–91.

[B54] Venu G (2014) Studies on silver staining of chromosomes of caecilians (Amphibia:Gymnophiona) of Western Ghats of India. International Journal of Advanced Research 2(3): 63–72.

[B55] Vitelli L Batistoni R Andronico F Nardi I Barsacchi-Pilone G (1982) Chromosomal localization of 18S + 28S and 5S ribosomal RNA genes in evolutionarily diverse anuran amphibians. Chromosoma 84: 475–491. https://doi.org/10.1007/BF00292849 7075349 10.1007/BF00292849

[B56] Wiley JE Little ML (2000) Replication banding patterns of the diploid-tetraploid treefrogs *Hyla chrysoscelis* and *H. versicolor*. Cytogenetics and Cell Genetics 88: 11–14. https://doi.org/10.1159/000015475 10773656 10.1159/000015475

[B57] Wiley JE Little ML Romano MA Blount DA Cline GR (1989) Polymorphism in the location of the 18S and 28S rRNA genes on the chromosomes of the diploid-tetraploid tree frogs *Hyla chrysoscelis* and *H. versicolor*. Chromosoma 97: 481–487. https://doi.org/10.1007/BF00295033

[B58] Zaleśna A Choleva L Ogielska M Rábová M Marec F Ráb P (2011) Evidence for integirty of parental genomes in the diploid hybridogenetic water frog *Pelophylax esculentus* by genomic in situ hybridization. Cytogenetic and Genome Research 134(3): 206–212. 21555873 10.1159/000327716

